# Potential relationship between eGFR_cystatin C_/eGFR_creatinine_‐ratio and glomerular basement membrane thickness in diabetic kidney disease

**DOI:** 10.14814/phy2.14939

**Published:** 2021-07-13

**Authors:** Carl M. Öberg, Martin Lindström, Anders Grubb, Anders Christensson

**Affiliations:** ^1^ Department of Clinical Sciences Lund Lund University Lund Sweden; ^2^ Department of Nephrology Skåne University Hospital Lund Sweden; ^3^ Department of Laboratory Medicine Malmö Lund University Malmö Sweden; ^4^ Center for Molecular Pathology Skåne University Hospital Malmö Sweden; ^5^ Department of Laboratory Medicine Lund University Lund Sweden; ^6^ Department of Clinical Chemistry Skåne University Hospital Lund Sweden; ^7^ Department of Clinical Sciences Malmö Lund University Lund Sweden; ^8^ Department of Nephrology Skåne University Hospital Malmö Sweden

**Keywords:** diabetic kidney disease, estimated GFR, glomerular barrier thickness, ratio eGFR_cystatin C_/eGFR_creatinine_, two‐pore model

## Abstract

Diabetic kidney disease (DKD) is a leading cause of end‐stage renal disease and renal replacement therapy worldwide. A pathophysiological hallmark of DKD is glomerular basal membrane (GBM) thickening, whereas this feature is absent in minimal change disease (MCD). According to fundamental transport physiological principles, a thicker GBM will impede the diffusion of middle‐molecules such as cystatin C, potentially leading to a lower estimated GFR (eGFR) from cystatin C compared to that of creatinine. Here we test the hypothesis that thickening of the glomerular filter leads to an increased diffusion length, and lower clearance, of cystatin C. Twenty‐nine patients with a kidney biopsy diagnosis of either DKD (*n* = 17) or MCD (*n* = 12) were retrospectively included in the study. GBM thickness was measured at 20 separate locations in the biopsy specimen and plasma levels of cystatin C and creatinine were retrieved from health records. A modified two‐pore model was used to simulate the effects of a thicker GBM on glomerular water and solute transport. The mean age of the patients was 52 years, and 38% were women. The mean eGFR_cystatin C_/eGFR_creatinine_‐ratio was 74% in DKD compared to 98% in MCD (*p* < 0.001). Average GBM thickness was strongly inversely correlated to the eGFR_cystatin C_/eGFR_creatinine_‐ratio (Pearson's *r* = −0.61, *p* < 0.01). Two‐pore modeling predicted a eGFR_cystatin C_/eGFR_creatinine_‐ratio of 78% in DKD. We provide clinical and theoretical evidence suggesting that thickening of the glomerular filter, increasing the diffusion length of cystatin C, lowers the eGFR_cystatin C_/eGFR_creatinine_‐ratio in DKD.

## Graphical abstract

Diabetes and chronic kidney disease is rapidly increasing globally. Here we found a correlation between eGFR_cystatin C_/eGFR_creatinine_‐ratio and glomerular basement membrane thickness in diabetic kidney disease. The results imply that a reduced eGFR_cystatin C_/eGFR_creatinine_‐ratio could be a possible biomarker for diabetic nephropathy.

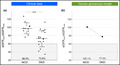

## 1 INTRODUCTION

The number of patients with diabetes and chronic kidney disease (CKD) is rapidly increasing worldwide (Liyanage et al., 2015). Advanced diabetic kidney disease (DKD) is considered an irrecoverable condition, and identifying diagnostic methods and applying early interventions are crucial to reduce the global burden of end‐stage renal disease.

Cystatin C has emerged as a marker for noninvasive estimation of GFR (Soveri et al., 2014) ever since it was noted that its reciprocal concentration correlated closely with GFR (Simonsen et al., 1985). Compared to creatinine, it appears to be especially well suited for the detection of early impairments in renal function (Newman et al., 1995; Shlipak et al., 2013). Cystatin C has a rather high molecular weight (MW) of 13.3 kDa and a Stokes‐Einstein (SE) radius of ~1.8 nm compared to that of smaller endogenous markers such as creatinine (MW 0.1 kDa and SE‐radius 0.3 nm). Nonetheless, cystatin C is regarded as a better predictor of adverse outcomes (Smith, 2013; Tangri et al., 2012) as well as a better marker for GFR in combination with creatinine (Dardashti et al., 2016; Dharnidharka et al., 2002) compared to creatinine alone.

The glomerular clearance of a small molecule like creatinine should very closely match that of the permeate flux (i.e., the glomerular filtration rate) over the glomerular filtration barrier (GFB), whereas the clearance of larger molecules will be increasingly dependent on the size of the selective elements in the GFB (i.e., the functional small pore radius). In fact, while most of the plasma content of cystatin C and other small plasma proteins are catabolized in the renal tubules (Tenstad et al., 1996), cystatin C is not freely filtered across the GFB (sieving coefficient ~0.84). Being a middle‐sized molecule it likely has a glomerular fractional clearance similar to that of β_2_‐microglobulin (Lund et al., 2003) and other small plasma proteins (Simonsen et al., 1985) (or 26 Å dextran [Oberbauer et al., 2000]).

Shrunken pore syndrome (SPS) is defined as a difference in estimated GFR from cystatin C versus creatinine (eGFR_Cystatin C_ <60% eGFR_Creatinine_ in the absence of non‐renal influence on the levels of cystatin C or creatinine [Grubb, 2020]), and has been identified as a strong independent risk factor for mortality in patients undergoing elective coronary artery bypass grafting (Dardashti et al., 2016) and CKD. This effect was seen also regardless of eGFR level (Åkesson et al., 2020). The suggested mechanism for SPS is a shrinking of the glomerular pore size leading to an increased plasma concentration of middle‐sized plasma proteins like cystatin C, β_2_‐microglobulin, beta‐trace protein, and retinol binding protein (Grubb, 2020; Grubb et al., 2015). Small plasma proteins like cystatin C are cleared from the body by glomerular filtration and degraded by renal tubular catabolism whereas only smaller amount of large proteins are cleared in the kidneys (Norden et al., 2001; Rippe & Öberg, 2016). Thus, when the glomerular filtration rate is decreased due to renal disease, plasma concentrations of small plasma solutes will increase (Grubb et al., 2015) which is the basis of noninvasive GFR estimation.

A decreased pore size is not the only possible mechanism leading to SPS and/or reduced estimated GFR from cystatin C versus creatinine, but could potentially result also from a thicker glomerular barrier leading to an increased diffusion length for middle molecules like cystatin C. One of the earliest structural alterations in DKD, preceding microproteinuria, is thickening of the glomerular basal membrane (GBM) (Marshall, 2016), whereas this condition is absent in minimal change disease (MCD). The aim of the current study was to test the hypothesis that estimated GFR from cystatin C is lower compared to that of creatinine in DKD versus MCD, and that this difference is correlated to GBM thickness. To explore if the observed alterations can be explained theoretically, we used the heteroporous two‐pore model by Deen et al. (1985) adapted to human physiological conditions. Due to the limited experimental information available on the hemodynamic conditions in the human glomerulus, some physiological parameters had to be estimated based on previous modeling by Oken (1982) and Navar et al. (2008).

## 2 METHODS

### 2.1 Design, setting, and participants

All patients referred for a kidney biopsy in Skåne, southern part of Sweden, are registered in a local data repository called the *Örestadsregistret* (*Örestad Registry*). We assessed 579 entries in the Örestad Registry between August 2013 and August 2017. Inclusion criteria were biopsy diagnosis of DKD or MCD and estimated GFR measurement using both cystatin C and creatinine within 2 weeks of the time of biopsy. All patients were assessed for medication including angiotensin‐converting enzyme inhibitors/angiotensin‐receptor blockers and corticosteroids. Exclusion criteria were signs of other pathologies in the biopsy specimen, for example, Ig A nephropathy or hypertensive nephrosclerosis. We identified 12 patients with MCD and 17 patients with DKD who fulfilled the inclusion criteria and none of the exclusion criteria. The Research Ethics Board at Lund University approved the study (Dnr 2017/568). Patient data and samples were treated anonymously in all statistical analyses.

### 2.2 Laboratory measurements for renal function

Plasma concentrations of creatinine and cystatin C were collected from health records. Cystatin C was determined by a particle‐enhanced immunoturbidimetric method using a reference material traceable to the international cystatin C calibrator (Grubb et al., 2014). Creatinine was determined by an enzymatic colorimetric assay using a calibrator traceable to primary reference material with values assigned by isotope dilution mass spectrometry (Nyman et al., 2014). GFR was estimated (eGFR) from the LMrev formula based on creatinine (Nyman et al., 2014) and the CAPA formula based on cystatin C (Grubb et al., 2014). These two equations are validated in Swedish populations.

### 2.3 A modified two‐pore model to simulate the effects of a thicker GBM on glomerular water and solute transport

Three pathophysiological changes will affect glomerular solute and water transport in DKD: (1) GBM hypertrophy, (2) glomerular hypertension, and (3) hyperfiltration (single nephron). We simulated these changes by (1) lowering the filtration coefficient (LpA) and solute diffusion capacity (half for twice the thickness and so on), (2) increasing the glomerular hydraulic pressure gradient to achieve, and (3) a single nephron GFR (SNGFR) of 90 nL/min. If the glomerular filtration coefficient, hydraulic pressure gradient, SNGFR, and (afferent) oncotic pressure are known, then the single nephron plasma flow profile *Q*(*y*) and the plasma protein concentration *C*_pr_(*y*) as functions of the relative position *y* (position/total length) along the glomerular capillary, can be determined by use of the conservation of mass equations

(1) $$C_{\text{pr}}\left(0\right)Q\left(0\right)=C_{\text{pr}}\left(y\right)Q\left(y\right),$$

(2) $$\frac{dQ\left(y\right)}{dy}=‐J_{v}\left(y\right).$$

Re‐arranging these equations gives the well‐known (Deen et al., 1972; Öberg et al., 2017; Thomson et al., 2007) non‐linear ordinary differential equation.

(3) $$\frac{dC_{\text{pr}}}{dy}=\frac{C_{\text{pr}}^{2}}{C_{\text{pr}}\left(0\right)Q\left(0\right)}J_{v}\left(y\right).$$

Above, *J*_v_(*y*) is the local filtration flux (Thomson et al., 2007) which is equal to the local Starling pressure (difference between hydraulic and oncotic pressure gradients, *P*_drop_ [*y*] is drop in hydraulic pressure across the length of the glomerular capillary) multiplied by the ultrafiltration coefficient (L_p_A).

(4) $$J_{v}\left(y\right)=L_{p}A\left(\left(P_{\text{GC}}‐P_{\text{BS}}‐P_{\text{drop}}\left(y\right)\right)‐\left(\Pi_{\text{GC}}\left(y\right)‐\Pi_{\text{BS}}\right)\right).$$

The colloid osmotic pressure in the glomerular capillary (Π_GC_) was calculated using the Landis–Pappenheimer equation, ($\Pi =2.1C_{\text{pr}}+0.16C_{\text{pr}}^{2}+0.009C_{\text{pr}}^{3}$), assuming a total plasma protein concentration of *C*_pr_(0) = 6.7 g/dl corresponding to a plasma colloid osmotic pressure of ~24 mmHg. In the human glomerulus, the capsular hydrostatic pressure (*P*_BS_) has been estimated to be between 15 and 20 mmHg (Navar et al., 2008; Oken, 1982) and the capsular oncotic pressure (Π_BS_) 0 mmHg. In order to achieve an effective filtration pressure (EFP) in the normal state of ~10 mmHg, we assumed a glomerular hydraulic pressure gradient of 40 mmHg (cf. Navar et al., 2008; Oken, 1982), implying a glomerular capillary pressure (*P*_GC_) of 55–60 mmHg. The pressure drop along the glomerular capillary network was set to 1 mmHg (Deen et al., 1972). The renal plasma flow (RPF or Q(0)) was determined with the gradient descent algorithm described in Öberg et al. (2017).

Actual measurements of glomerular sieving coefficients for small proteins like cystatin C across the human glomerular filter are scarce in the literature. Perhaps the most reliable estimations are those obtained in children with Fanconi syndrome (Edwards et al., 2020; Norden et al., 2001), although these sieving coefficients may be slightly low due to residual tubular reabsorption (Edwards et al., 2020). Based on the sieving data in Norden et al. (2001) and Lund et al. (2003) and previous results from the rat GFB (Oberg & Rippe, 2014) we assumed a small pore radius (*r*_S_) of 3.7 nm and a large pore radius (*r*_L_) of 11.0 nm (see also Lund et al., 2003; Oberg & Rippe, 2014). Again, if assuming conservation of mass (see also Öberg et al., 2017), the change in solute concentration *C*_i_(*y*) due to glomerular filtration (at position *y* along the glomerular capillary) is given by

(5) $$\frac{dC_{i}}{dy}=‐\frac{J_{s}\left(y\right)‐J_{v}\left(y\right)C_{i}}{Q\left(y\right)},$$

where the local solute flux of a solute, *J*_s_(*y*), is calculated from

(6) $$J_{s}\left(y\right)=f_{S}J_{v}\left(y\right)\frac{C_{i}\left(y\right)W_{S}}{1‐\left(1‐W_{S}\right)e^{‐{\text{Pe}}_{S}}}+f_{L}J_{v}\left(y\right)\frac{C_{i}\left(y\right)W_{L}}{1‐\left(1‐W_{L}\right)e^{‐{\text{Pe}}_{L}}},$$

where *C*_i_(*y*) is the solute concentration at position *y* along the glomerular capillary; *f*_S_ and *f*_L_ are the fractional volume flows; *W*_S_ and *W*_L_ are the convective hindrance factors (Dechadilok & Deen, 2006), across the small‐ and the large pores respectively. Equation 6 was used for all solute sizes in the simulations. For details regarding the numerical solutions of the above equations, see Öberg et al. (2017). Péclet numbers (Pe_S_ and Pe_L_) are calculated from

(7) $${\text{Pe}}_{S}=f_{S}J_{v}\left(y\right)\frac{W_{S}}{{\text{PS}}_{S}},$$

(8) $${\text{Pe}}_{L}=f_{L}J_{v}\left(y\right)\frac{W_{L}}{{\text{PS}}_{L}}.$$

Solute diffusion capacities PS_S_ and PS_L_ (cf. Equations 8 and 9 in Deen et al. (1985)) were calculated from

(9) $${\text{PS}}_{S}=H_{S}D\cdot \alpha_{S}L_{p}A\frac{8\eta }{r_{S}^{2}},$$

(10) $${\text{PS}}_{L}=H_{L}D\cdot \alpha_{L}L_{p}A\frac{8\eta }{r_{L}^{2}},$$

where *H*_S_ and *H*_L_ are the diffusive hindrance factors (Dechadilok & Deen, 2006), *D* is the free diffusion coefficient calculated from the Stokes–Einstein radius of the solute protein, η is the viscosity of the permeate (0.7 mPa∙s), *α*_S_ and *α*_L_ denote the fractional hydraulic conductance of the small and large pore system, respectively (for a discussion on the differences between *α*_S/L_ and *f*_S/L_ and their calculation, see Oberg & Rippe, 2014). In the current article, the *f*_L_ parameter was set to ~1 × 10^−4^ (similar to that obtained in humans using Ficoll [Blouch et al., 1997]) which gives a *α*_L_ of ~3 × 10^−5^. We assumed a steady‐state plasma clearance of cystatin C (and creatinine), occurring chiefly via glomerular filtration:

(11) $${\text{Cl}}_{\text{cysC}}=\text{GFR}\cdot \theta_{\text{cysC}},$$

where θ_cysC_ is the glomerular sieving coefficient of cystatin C. A small pore system with a 3.7‐nm pore radius implies a sieving coefficient for cystatin C of ~0.87. For an adult male having a GFR of 120 ml/min, this translates to a cystatin C concentration of about 0.69 mg/L.

### 2.4 Measurement of GBM

Core biopsies were first put in 4% formaldehyde and thereafter embedded in paraffin. Cases were sectioned in two levels, each 1.5 µm thick, and stained for hematoxylin and eosin staining, Alcian blue periodic acid‐schiff, Trichrome staining, elastin van Gieson staining, periodic Schiff‐methenamine silver, alkaline Congo red, and each case were re‐evaluated by light microscopy. Samples for electron microscopy (EM) were put in 4% formaldehyde and embedded in (plastic) and thereafter sectioned in 60 nm thick sections. Transmission EM was performed for each case with a transmission microscope (TECNAI FEJ Company) as well as photo documented in JPEG‐format by a digital camera (VELETA). One glomerulus per case with at least one full segment was examined with EM. It is standard in clinical practice to examine 1 or 2 glomeruli for EM while the rest is embedded in paraffin for light microscopy. Each case was evaluated regarding basal membranes, foot processes, and mesangium and were found to correspond to the diagnosis of diabetic nephropathy or minimal change nephropathy. Each case showed homogenous thickness of basal membranes of segments without any focal irregularities or deposits. For each glomeruli, 20 measurements points were taken in a representative loop. GBM thickness was assessed through ultrastructural morphometry on enlarged electron micrographs as the arithmetic mean of 20 orthogonal intercepts across the GBM. Electron micrographs were available in 8 patients with MCD and in 11 patients with DKD.

### 2.5 Statistical methods

Values are presented as average (IQR) unless otherwise specified. Differences between groups were assessed using a Wilcoxon signed rank test. Monte Carlo‐based power analysis (10,000 simulations) was conducted, applying Wilcoxon tests between fixed unequal sample sizes of 17 and 12 patients, showing that the study had 89% power to detect a true difference of 0.2 in eGFR_cystatin C_/eGFR_creatinine_‐ratio assuming a SD of 0.16 (Cohen's *d* = 1.25). Moreover, a sample size of 19 patients had 87% power to detect a true correlation coefficient of 0.6 or better between GBM‐thickness and eGFR_cystatin C_/eGFR_creatinine_‐ratio, at a significance level of *p* < 0.05. A post‐hoc ANCOVA was applied using the model (ratio ~eGFR + diagnosis) using type III SS. We assumed an α‐level of 0.05 and a β‐level of 0.20 unless otherwise specified. *p*‐values are denoted: * for *p* < 0.05, ** for *p* < 0.01 and *** for *p* < 0.001. All statistical calculations were performed using R for macOS (R Foundation for Statistical Computing, version 3.5.1).

## 3 RESULTS

### 3.1 Reduced ratio of eGFR_cystatin C_/eGFR_creatinine_ in DKD versus MCD

**FIGURE 1:**
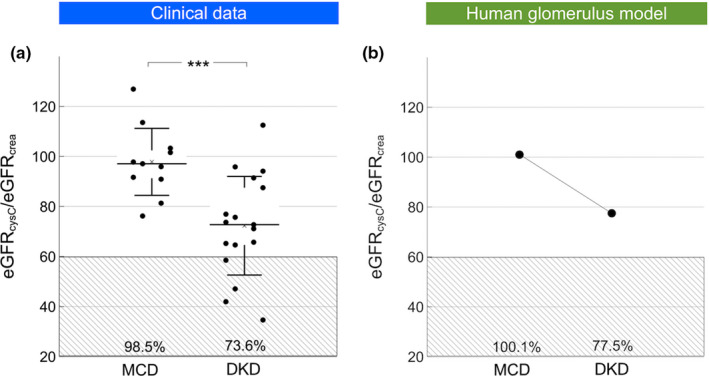
(a) Estimated GFR from cystatin C and creatinine ratio in patients with a biopsy diagnosis of either diabetic kidney disease (DKD; *n* = 17) or minimal change disease (MCD; *n* = 12). (b) Estimated GFR from cystatin C and creatinine in a theoretical model of the human glomerular filtration barrier. In silico simulations were performed either assuming a thickness of the glomerular basement membrane (GBM; 300 nm) from a glomerulus from a patient with MCD or a hypertrophic GBM being three times thicker (900 nm) from patient with DKD. It was assumed that GFR remained constant as a result of tubuloglomerular feedback, thus implying a three times higher effective filtration pressure across the glomerulus in the hypertrophic condition. *** *p* < 0.001.

We first assessed estimated GFR (eGFR) from cystatin C and creatinine in two groups of patients with a biopsy diagnosis of either DKD (*n* = 17) or MCD (*n* = 12). Baseline characteristics and eGFR estimations are shown in Table 1. Median age of the patients was 52 (41–64) years at the time of biopsy with no difference between the groups, and 38% were female. Plotted in Figure 1a is the ratio eGFR_cystatin C_/eGFR_creatinine_ which was lower (*p* = 0.0004, *W* = 26) in DKD patients compared with MCD patients. Furthermore, four patients with DKD also fulfilled the criteria for SPS, defined as eGFR_cystatin C_ <60% eGFR_creatinine_ whereas none of the MCD patients fulfilled this criteria (the lowest ratio in the MCD group was ~76%). Average estimated GFR (eGFR), calculated as the arithmetic average of eGFR_cystatin C_ and eGFR_creatinine_, was different in the groups, which is expected since GFR is usually much less affected in MCD (Table 1). To investigate if reduced eGFR was in itself correlated to a reduced ratio, we performed a post hoc analysis of co‐variance showing no significant main effect of eGFR on the eGFR_cystatin C_/eGFR_creatinine_‐ratio (*F*_1,26_ = 0.2, *p* = 0.65).

**TABLE 1:** Clinical GFR estimations

Parameter	DKD (*n* = 17)	MCD (*n* = 12)
Age, years	56 (43–65)	46 (30–54)^†^
eGFR_crea_,^a^ (ml/min/1.73 m^2^)	32 (21–38)	88 (48–97)***
eGFR_cysC_,^b^ (ml/min/1.73 m^2^)	23 (14–27)	73 (46–96)***
eGFR^c^, (ml/min/1.73 m^2^)	25 (18–32)	82 (47–96)***
GBM thickness, (nm)	747 (620–839)	376 (335–404)***
U‐alb/creat ratio (mg/mmol)	320 (1–419)	85 (1–557)^††^
ACEi/ARB use (*n*)	9	0
Corticosteroid use (*n*)	0	3

Values are given as median (IQ range) and measured at time of biopsy.

Abbreviations: DKD, diabetic kidney disease; GBM, glomerular basal membrane; GFR, estimated GFR; MCD, minimal change disease; *n*, number of patients.

^a^ Calculated using the CAPA equation (Grubb, 2020): eGFR_CAPA_ = 130 × cystatin C^−1.069^ × age^−0.117^–7.

^b^ Calculated using the LM rev equation (Grubb et al., 2014). $e^{X‐0.0158\times \text{age}+0.438\times \ln(\text{age})}$: Female pCr <150 μmol/L: *X* = 2.50 + 0.0121 × (150–pCr); Female pCr ≥150 μmol/L: *X* = 2.50–0.926 × ln(pCr/150); Male pCr <150 μmol/L: *X* = 2.56 + 0.00968 × (180–pCr); Male pCr <150 μmol/L: *X* = 2.56–0.926 × ln(pCr/180).

^c^ Average estimated GFR.

^†^ *p* = 0.13.

^††^ *p* = 0.23.

*** *p* < 0.001.

### 3.2 The eGFR_cystatin C_/eGFR_creatinine_‐ratio is strongly correlated to GBM thickness

**FIGURE 2:**
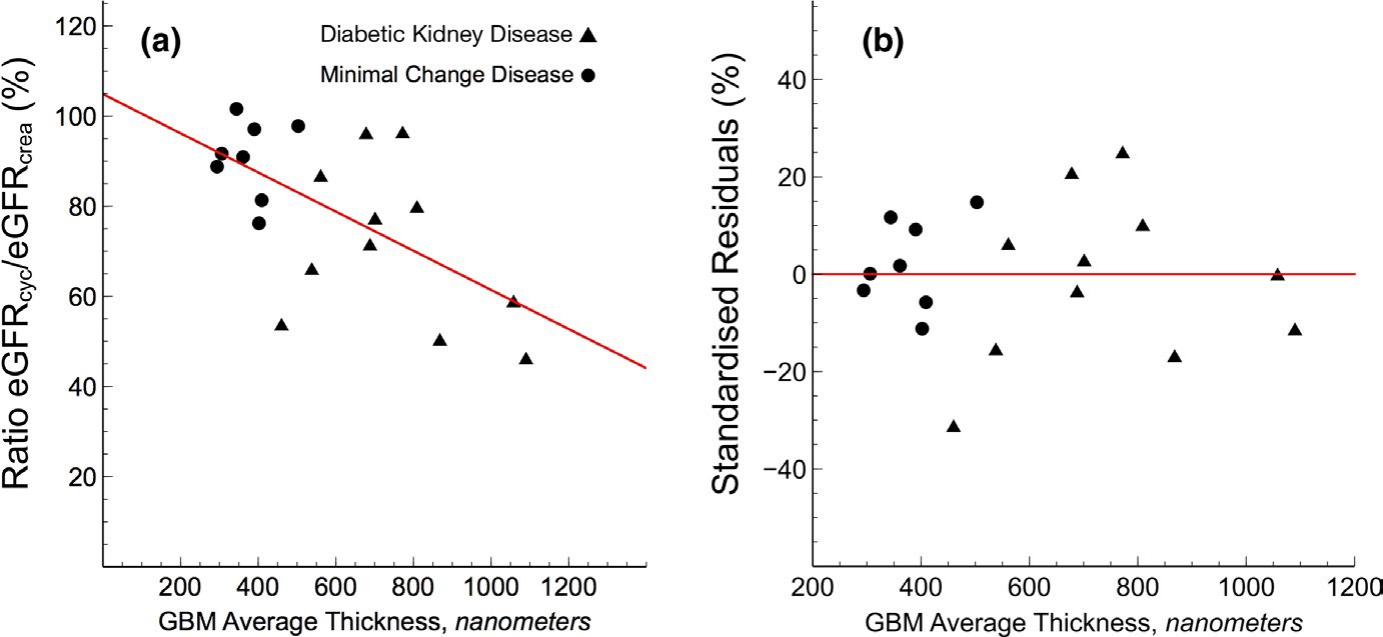
(a) Estimated GFR from cystatin C and creatinine (eGFR_cystatin C_/eGFR_creatinine_) ratio versus glomerular basement membrane thickness as assessed via electron micrographs from kidney biopsies. (b) Difference between modeled and measured eGFR_cystatin C_/eGFR_creatinine_‐ratio as a function of glomerular basement membrane thickness

Linear regression was performed to investigate the theoretically predicted relationship between the eGFR_cystatin C_/eGFR_creatinine_‐ratio and GBM thickness (nm). The scatterplot (Figure 2) indeed suggested a negative linear relationship between the two, which was confirmed with a Pearson's correlation coefficient of −0.61. Simple linear regression showed a significant relationship between eGFR_cystatin C_/eGFR_creatinine_‐ratio and GBM thickness (nm) (*p* < 0.01). The slope coefficient for mean GBM‐thickness was −0.04% nm^−1^ so the eGFR_cystatin C_/eGFR_creatinine_‐ratio was apparently reduced by ~4% for every 100 nm increased thickness. As shown in Figure 2b, the plot of residuals versus the independent parameter indicated that the residuals were approximately normally distributed. To explore whether body composition and muscle mass affect the ratio eGFR_cystatin C_/eGFR_creatinine_ we used linear correlation between weight versus eGFR_cystatin C_/eGFR_creatinine_‐ratio and body mass index (BMI) versus eGFR_cystatin C_/eGFR_creatinine_‐ratio, but found no significant association.

### 3.3 Reduced ratio of eGFR_cystatin C_/eGFR_creatinine_ is theoretically dependent on GBM thickness

**FIGURE 3:**
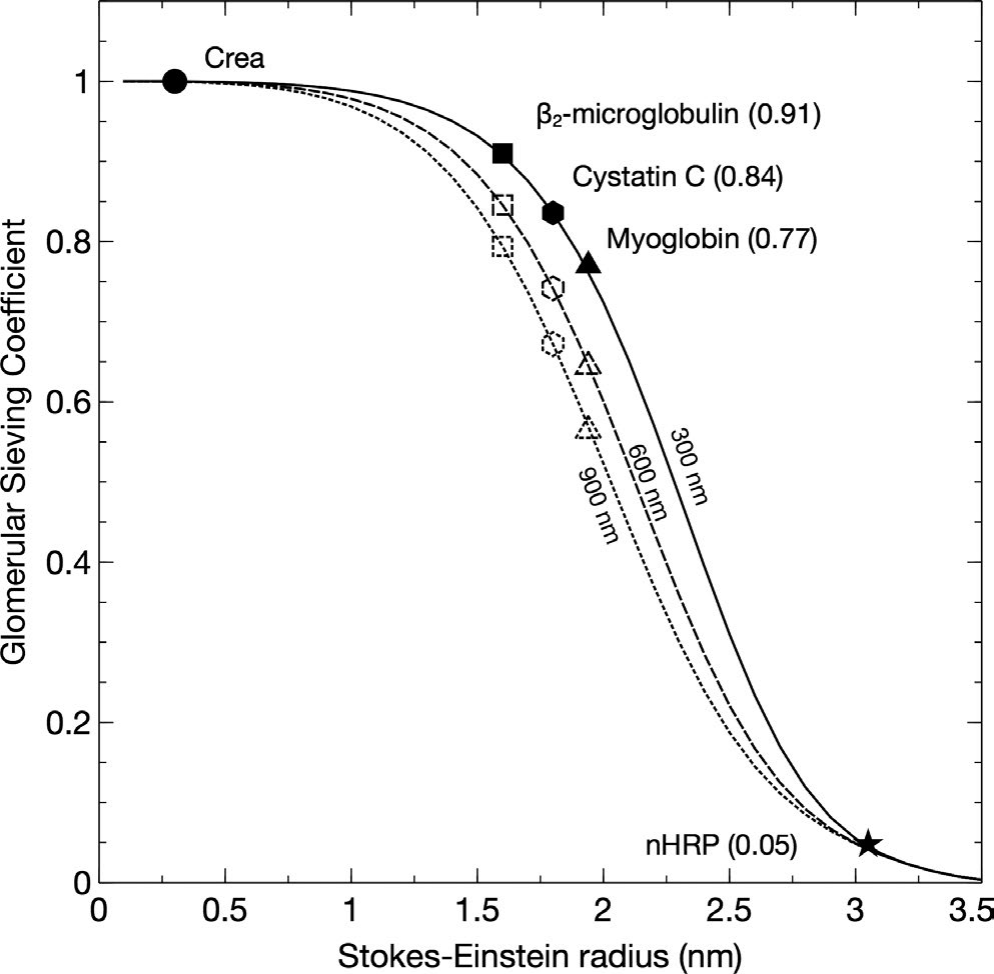
Glomerular sieving coefficients versus the Stokes‐Einstein radii of the solute proteins for three different thicknesses of the glomerular basement membrane 300 nm (and an effective filtration pressure [EFP] of 10 mmHg), 600 nm (EFP 20 mmHg), 900 nm (EFP 30 mmHg). Also shown are experimentally measured sieving coefficients for β_2_‐microglobulin (Norden et al., 2001) (black square) and myoglobin (Lund et al., 2003) (black triangle). The sieving coefficient of a 3.05 nm protein (neutral horseradish peroxidase nHRP [Lund et al., 2003]; solid star) will be practically unaltered by the thickening of the renal filter

Pathophysiological hallmarks in the development of diabetic glomerulosclerosis are glomerular hypertrophy, hypertension, and hyperfiltration; processes that were here modeled in silico by increasing the thickness of the GBM and the EFP to result in a SNGFR of 90 nL/min. We first increased glomerular pressure linearly with decreasing GFB conductance (+10 mmHg for twice the thickness, +20 mmHg for triple, and so on). This had the effect of reducing the eGFR_cystatin C_/eGFR_creatinine_ ratio. Glomerular hypertension and GBM hypertrophy evoked marked reductions in the clearance of cystatin C (Figures 1b and 3). Plotted in Figure 1b are the simulated ratios of eGFR_cystatin C_/eGFR_creatinine_ in a normal glomerulus having a glomerular basement membrane thickness of 300 nm and an EFP of 10 mmHg versus a “diabetic” glomerulus having a glomerular basement membrane thickness of 900 nm and an EFP of 30 mmHg. According to the current model, the impact of differently sized solutes is heterogeneous. Shown in Figure 3 are simulated glomerular sieving coefficients versus the Stokes–Einstein radii of the solute proteins for three different scenarios. The solid line represents a normal healthy subject having a glomerular basement membrane thickness of ~300 nm and an EFP of 10 mmHg. Also plotted are experimentally measured sieving coefficients for β_2_‐microglobulin (Norden et al., 2001) (black square) and myoglobin (Lund et al., 2003) (black triangle). The simulated sieving coefficient of cystatin C is also shown (black hexagon). The sieving coefficient for cystatin C is, under normal conditions ~0.84 corresponding to a glomerular clearance of ~5/6 of GFR, that is, Cl_CC_ ≈100 ml/min/1.73 m^2^ for a relative GFR of 120 ml/min. For the scenarios with a thicker glomerular filter (dashed and dotted line), glomerular hypertension is assumed necessary to maintain the same GFR and the EFP will increase linearly with the thickness of the barrier, thus for a 600 nm barrier (dashed line) the EFP is 20 mmHg. This thickening of the barrier will also double the diffusion distance for cystatin C, effectively lowering its fractional clearance to 0.74 (Cl_CC_ ≈89 ml/min/1.73 m^2^) for the 600 nm barrier and to 0.67 for the 900‐nm barrier (Cl_CC_ ≈80 ml/min/1.73 m^2^). Assuming a constant production of cystatin C, these latter scenarios will cause the serum concentration of cystatin C (and other small proteins) to increase so that the estimated GFR using, for example, the CAPA equation (Grubb et al., 2014) will be lower than GFR. This effect is shown in Table 2. By contrast, the sieving coefficient of a 3.05‐nm protein (here exemplified by neutral horseradish peroxidase nHRP [Lund et al., 2003]; solid star) will be practically unaltered by the thickening of the renal filter.

**TABLE 2:** Glomerular hypertension and hypertrophy in a human glomerulus model

Barrier thickness	300 nm	600 nm	900 nm
GFR, ml/min/1.73 m^2^	120	120	120
Cystatin C, mg/L	0.70	0.79	0.88
eGFR_CAPA_ ^a^	121	105	93

Barrier thickness	300 nm	600 nm	900 nm
GFR, ml/min/1.73 m^2^	120	120	120
Cystatin C, mg/L	0.70	0.79	0.88
eGFR_CAPA_ ^a^	121	105	93

^a^ eGFR_CAPA_ = 130 × cystatin C^−1.069^ × age^−0.117^−7.

### 3.4 Theoretical effects of actual “shrunken pores” in the glomerular filter

**FIGURE 4:**
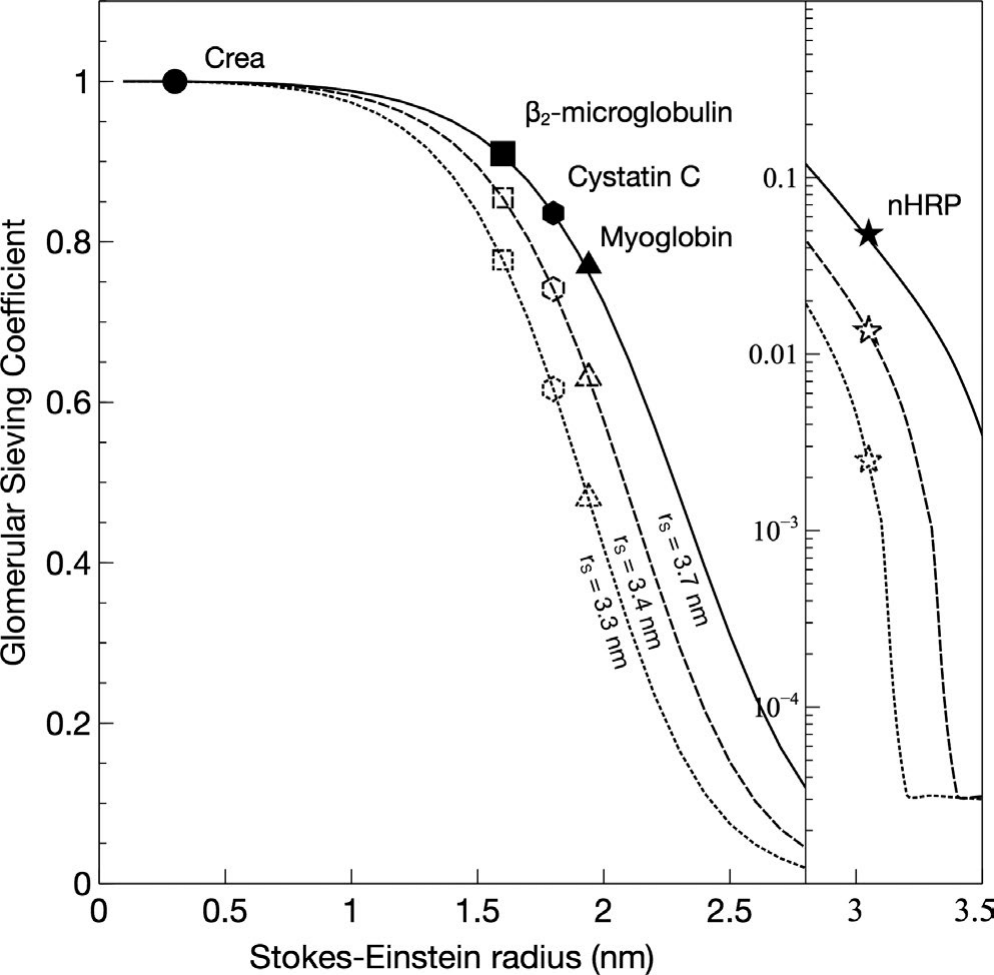
Glomerular sieving coefficients versus Stokes–Einstein radius for scenarios with shrunken pores, 3.4 nm (dashed line), and 3.2 nm (dotted line) compared to normal (3.7 nm; solid line)

**FIGURE 5:**
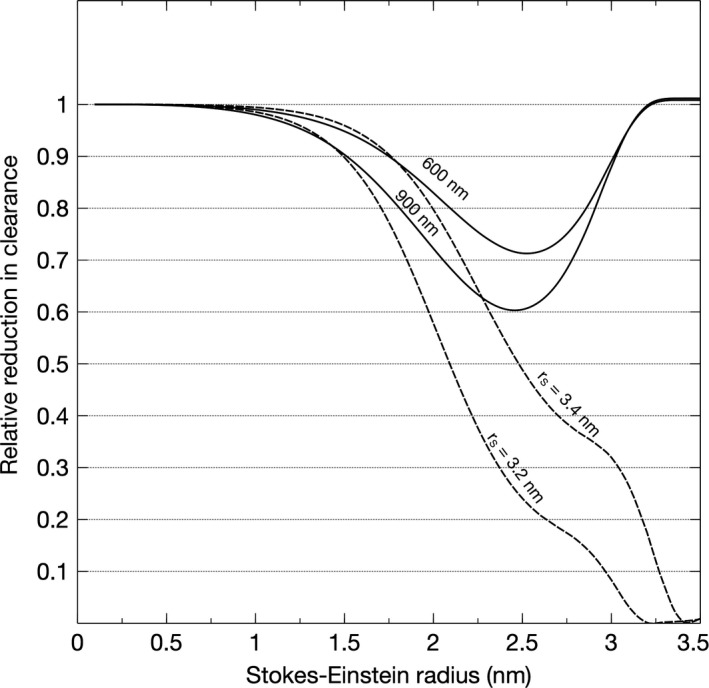
Pathological versus healthy scenarios, either due to thickening of the glomerular filter (solid lines) to 600 or 900 nm, or shrinking of the functional pores (dashed lines) to 3.4 or 3.2 nm

In Figure 4, the modeled sieving coefficients versus SE‐radius are plotted for scenarios with shrunken pores, 3.4 nm (dashed line), and 3.2 nm (dotted line). Expectedly, shrinking the pore size causes the sieving curve to shift to the left. As can be seen, pore shrinking has similar effects to thickening of the GFB for smaller proteins. However, by contrast, it has marked effects for larger solutes having radii close to the pore radius (<3.7 nm). For the 3.05‐nm protein (nHRP, star), the sieving coefficient will be reduced by a factor ~20 if the pores are shrunken to 3.2 nm. The effects of the different scenarios are demonstrated in Figure 5, where the ratio of the pathological versus healthy scenarios in Figures 3 and 4 are plotted. Thus, an ideal solute size to detect GFB thickening would be approximately 2.5 nm whereas solutes exceeding 3.0 nm would be essentially unaffected under the current assumptions of an unchanged GFR, but the same effect will be present during conditions with a lower GFR. Actually, for slightly decreased GFRs, CrCl will typically overestimate the actual GFR contributing to SPS.

## 4 DISCUSSION

We here provide theoretical modeling and clinical data suggesting that the diffusive clearance of middle‐molecular species such as cystatin C is reduced in DKD to a degree corresponding with the increment in the thickness of the GBM. We demonstrate an inverse linear relationship between the ratio eGFR_cystatin C_/eGFR_creatinine_ and basal membrane thickness. According to theory, during conditions of glomerular vascular wall thickening, there should be a measurable difference in the eGFR measured using cystatin C and the actual GFR (e.g., as measured by iohexol) under the assumption that their plasma concentrations are determined chiefly by glomerular filtration. In line with our present results, a decreased diffusional clearance of mid‐sized molecules has been found in experimental and clinical studies of DKD (Lubbad et al., 2015; Oberbauer et al., 2000; Scandling & Myers, 1992). There were; however, considerable variations in the eGFR_cystatin C_/eGFR_creatinine_ ratio in our data indicating that factors other than glomerular filtration may be involved. It is well‐known that the steady‐state plasma concentration of creatinine is affected by a number of other factors (Perrone et al., 1992) which limits its use as a reliable GFR‐marker. Similarly, cystatin C concentrations are affected by conditions such as high‐dose corticosteroid medication (Cimerman et al., 2000). A limitation in the current study is therefore that it cannot be excluded that factors other than glomerular filtration affected the plasma levels of cystatin C and/or creatinine. A low muscle mass could give a higher eGFR_creatinine_ and thus lower eGFR_cystatin C_/eGFR_creatinine_ ratio. Regarding the effect of muscle mass on SPS we could not find a correlation between BMI and eGFR_cystatin C_/eGFR_creatinine_ ratio. It has been described increased levels of cystatin C after high‐dose corticosteroids which would give a low eGFR_cystatin C_/eGFR_creatinine_ ratio. In our cohort only three patients with MCD were treated with at least 30 mg prednisolon at the time of biopsy and no patients with DKD, which rules out an effect of steroids on our results. Recently the SPS was reported which is characterized by a low ratio eGFR_cystatin C_/eGFR_creatinine_, and we wanted to study this ratio in correlation to the thickness of the basal membrane. However, the question of whether SPS is the result of actual shrunken pores or vascular wall hypertrophy cannot be determined from the current data. Theoretically, shrinking of pores has more dramatic effects on the renal clearance of larger middle‐molecules between 3.0 and 3.5 nm compared to small molecules, like creatinine and urea. Interestingly, many important plasma proteins, enzymes, and peptide hormones are in this size range (e.g., cardiac Troponin T, TSH, AST, ALT, and albumin) but many of them are catabolized in other tissues and regulated via other mechanisms making it unclear what effect shrunken pores would have on their plasma concentrations. It is well established that GFR is an important determinant of plasma cardiac troponin T (cTnT) levels (Abbas et al., 2005; Tsutamoto et al., 2009). According to our calculations, plasma cTnT levels should be several fold higher in a patient with SPS (~20 times higher for the 3.2 nm pore size), if SPS is caused by an actual "pore shrinkage".

The biselective nature of the GFB is well described in the literature (Oberg & Rippe, 2014). For the renal glomerular microcirculation, the first theoretical description was introduced by Deen et al. (1985) using a model consisting of two distinct pathways for solute transport. Thus far, the most "accurate" theoretical description of the GFB is similar to that of a highly size‐selective mechanical cross‐flow filter, meaning that most of the blood flow through the glomerular capillaries travels in a direction tangentially across the capillary wall rather than entering the renal filter. Also, the red blood cells travel through the capillary in a compressed “cell‐by‐cell fashion” and thus constantly comb the filter surface. Both of these mechanisms, tangential solvent flux, and continuous combing of the endothelial glycocalyx by RBCs, may counteract clogging of the renal filter. However, inevitably, some solutes must enter the capillary wall and, thus, must be dealt with via other mechanisms. Podocytes have been shown to exhibit endocytosis (and transcytosis) of albumin which will contribute to the clearance of albumin from the glomerular filter (Castrop & Schiessl, 2017).

To understand why only solutes between 1.0 and 3.0 nm are affected by a thicker GFB one must first understand the concept of diffusion capacity, the proportionality coefficient in Fick's equation

$$J_{s}=\text{PS}\cdot \Delta c.$$

Here PS is the diffusion capacity in ml/min, *J*_s_ is the diffusive solute flux in mmol/min and Δ*c* is the concentration gradient (Plasma water concentration−Bowman's space concentration) in mmol/ml. From the equation above, it can be seen that the diffusion capacity represents the maximal possible absolute diffusive clearance of a solute, being approached as the concentration on one side of the barrier is approaching zero. Mathematically, PS is the product of the diffusion coefficient *D* and the effective surface area *A* of the membrane divided by the membrane thickness Δ*x* (i.e. PS = D × *A*/Δ*x*), which means that PS will be reduced if the thickness, or diffusion length, Δ*x*, is increased. Due to their small size, solutes like creatinine, urea, glucose et cetera have a great capacity to be transported via diffusion over the GFB since they have a very large diffusion capacity. Yet, diffusion is a process that requires a concentration gradient; and since small solutes <1.0 nm are not sieved at all by the GFB Δ*c* = 0 they will be transported entirely via convection and their clearance will equal GFR. For mid‐sized solutes 1.0–3.0 nm, there is a small but important concentration difference across the renal filter, leading to a considerable part of their clearance being due to diffusion due to the fact that the diffusion capacity is still very high (>> higher than GFR) for these solutes. With increasing solute sizes >3.0 nm the diffusion capacity rapidly drops to low values (<< lower than GFR), and the diffusive clearance becomes negligible.

### 4.1 Strengths

A validated registry for renal biopsy in which GFR was estimated from both creatinine and cystatin C is a strength of this study. The ultrastructural morphometry of the thickness of the GBM was evaluated by an experienced pathologist on enlarged electron micrographs as the arithmetic mean of 20 orthogonal intercepts across the GBM.

### 4.2 Limitations

This study has several limitations. A major limitation is the low number of patients in both groups. However, the differences between the two groups, regarding eGFR_cystatin C_/eGFR_creatinine_ ratio and GBM thickness, were statistically significant. It would also be an advantage having more patients with early DKD. Sweden has a homogenous racial distribution which limits our conclusions to this population. We also used a very simplistic pore model for glomerular filtration during DKD, assuming that the hydraulic conductance (LpA) and solute diffusion capacities (PS) are inversely proportional to the thickness of the GBM. It is likely that the actual changes in these parameters are more complex. However, the lack of more accurate measurements of these parameters in patients with (or without) DKD, indicates that a more sophisticated approach is difficult to justify at the moment.

In conclusion DKD develops insidiously over several years, well before any clinical manifestations (e.g., declining GFR and microproteinuria) are evident, and once microproteinuria is present, structural lesions are often considerably advanced. Caramori et al. (2013) analyzed 94 biopsies in normoalbuminuric patients, and found that GBM thickness was the only structural parameter that predicted progression to diabetic nephropathy. Our data show that basal membrane thickness is associated with the clearance of freely filtered molecules in relation to their molecular weight obvious from a reduced ratio eGFR_cystatin C_/eGFR_creatinine_.

In light of our current findings, further research should explore the possibility that plasma levels of mid‐sized molecules like cystatin C could act as biomarkers for detecting DKD, allowing early treatment. The ratio eGFR_cystatin C_/eGFR_creatinine_ may also be helpful in diagnosing other cases of CKD in early phases. In this context, it should be pointed out that kidney diseases may result in both thickening and thinning of the GBM.

## DISCLOSURES

All the authors declare no conflict of interest.

## AUTHOR CONTRIBUTIONS

CAÖ and AC designed the study and collected data. CAÖ performed mathematical modeling. CAÖ and AC analyzed the data. CAÖ made the figures. ML analyzed kidney biopsies. CAÖ, AG, and AC drafted the manuscript. All authors contributed to, and approved the final version of the manuscript.
